# Differential Expression of miR-136 in Gestational Diabetes Mellitus Mediates the High-Glucose-Induced Trophoblast Cell Injury through Targeting E2F1

**DOI:** 10.1155/2020/3645371

**Published:** 2020-10-20

**Authors:** Chunxia Zhang, Li Wang, Jinfeng Chen, Fei Song, Yuzhen Guo

**Affiliations:** ^1^Department of Obstetrics and Gynecology, Shengli Oilfield Central Hospital, Dongying, Shandong 257000, China; ^2^Delivery Room, Shengli Oilfield Central Hospital, Dongying, Shandong 257000, China; ^3^Department of Obstetrics, Shengli Oilfield Central Hospital, Dongying, Shandong 257000, China; ^4^Clinical Laboratory, Shengli Oilfield Central Hospital, Dongying, Shandong 257000, China

## Abstract

**Background:**

Gestational diabetes mellitus (GDM) seriously affects the health of mothers and infants. The high-glucose-induced inhibition in trophoblast cell viability is an important event in GDM pathogenesis. This study evaluated the expression and clinical significance of miR-136 in GDM patients, and the biological function and related mechanisms of miR-136 in the regulation of trophoblast cell proliferation were explored.

**Methods:**

The expression of miR-136 in serum and placenta of GDM patients was measured using quantitative Real-Time PCR. Trophoblast cells were stimulated with high-glucose medium to mimic the pathological changes of GDM, and the effect of miR-136 was examined by CCK-8 assay. A luciferase reporter assay was used to confirm the target gene of miR-136, and the relationship of E2F transcription factor 1 (E2F1) with miR-136 in GDM was further analyzed.

**Results:**

miR-136 expression was significantly elevated in GDM serum and tissue samples. By high-glucose treatment, trophoblast cell proliferation was inhibited and miR-136 expression was promoted. The knockdown of miR-136 could promote the proliferation of trophoblast cells exposed to high glucose, whereas the overexpression of miR-136 could suppress it. In addition, E2F1 was identified as a target gene of miR-136, which could mediate the regulatory effect of miR-136 on trophoblast cell proliferation.

**Conclusion:**

Collectively, miR-136 expression is increased in both serum and placental tissues in GDM patients, and miR-136 mediates the inhibiting effect of high glucose on trophoblast cell viability by targeting E2F1.

## 1. Introduction

Gestational diabetes mellitus (GDM) is the first reduction with different degrees of impaired glucose appearing during pregnancy [1]. It is the most common medical complication in pregnancy and seriously affects the health of mothers and infants [2]. If GDM is not treated in time, it can cause a series of serious effects, such as fetal developmental abnormalities, abortion, and fetal asphyxia [3, 4]. Thus, timely diagnosis and treatment of GDM are needed. However, effective screening and diagnosis strategies for GDM are lacking in the world [5]. Studies have shown that fetal dysplasia may be related to the inhibitory effect of high-glucose environment on trophoblast cells. Inhibition of trophoblast cell proliferation activity will promote the poor formation of placental tissue, and placental tissue as a place for maternal-fetal nutrient exchange is essential for fetal growth and development. Therefore, the establishment and maintenance of a successful pregnancy mainly depend on trophoblast cells [6]. Exploring the treatment of GDM can start by improving the proliferative activity of trophoblast cells.

MicroRNAs (miRNAs) is noncoding RNA, 19-22 nucleotides, which is closely related to various diseases such as cardiovascular disease, cancer, and GDM. Studies have shown that the placenta contains a large amount of miRNA; miRNAs play an important role in regulating the behavior of trophoblast cells in the placenta. miRNAs can promote the proliferation of trophoblast and play a role in the pathogenesis of repeated abortions by interfering with the normal activity of trophoblast cells [7, 8]. At present, miR-136 plays a role in various diseases, such as lung squamous cell carcinoma [9], spinal cord injury [10], and stem cell cancer [11], which exhibits a regulatory effect on cell proliferation. Of note, in a recent study by Gillet et al., miR-136 has been documented to aberrantly express in patients with GDM [12]. However, it remains unclear whether the deregulated miR-136 had any clinical and biological significance in GDM progression.

E2F transcription factor 1 (E2F1) has been previously documented as a direct target of miR-136 in cervical carcinoma, and the miR-136/E2F1 axis acted important regulatory effects on cell proliferation [13]. E2F1 is first identified member of the E2F family, which serves as an important regulator of cell cycle [14]. Some studies have reported the biological function of E2F1 in the progression of diabetes and related complications [15, 16]. In GDM, E2F1 has been stated as a potential target of miR-330-3p, which was upregulated in GDM and related with disease development [17]. We also predicted the putative binding site of miR-136 at the 3′-UTR of E2F1 and sought to explore the relationship between miR-136 and E2F1 in trophoblast cells.

This study is aimed at exploring the expression of miR-136 in patients with GDM and analyzing its biological work in GDM progression. In addition, the molecular mechanisms underlying the functional role of miR-136 were explored by analyzing its relationship with E2F1, which may help to further understand the pathogenesis of GDM.

## 2. Materials and Methods

### 2.1. Patients and Sample Collection

All samples were collected from 112 patients with GDM and 58 health pregnant women in Shengli Oilfield Central Hospital from 2013 to 2018. The GDM patients in this study were diagnosed according to the standards recommended by the American Diabetes Association [18]. None of the GDM patients had pregestational diabetes, multiple gestation, or medication. Venous blood samples were collected from the participants at the 24–28 pregnancy weeks after an overnight fast, and serum samples were extracted. Fasting blood glucose (FBG) was measured using the glucose oxidase method. Placental tissues of the enrolled pregnant women were obtained at the time of delivery and stored with liquid nitrogen. The experimental procedures were approved by the Ethics Committee of Shengli Oilfield Central Hospital, and all the participants were informed and provided a paper-based form of informed letter.

### 2.2. Cell Culture and High-Glucose Treatment

Two human trophoblast cell lines HRT-8/SVneo and BeWo were purchased from the Type Culture Collection of the Chinese Academy of Sciences (Shanghai, China). HRT-8/SVneo was cultured in RPMI-1640 medium (BioTek Corporation, Beijing, China), and BeWo cell lines were cultured in Ham's F-12K medium (Gibco, CA, USA), which were added with 10% fetal bovine serum (FBS; Thermo Fisher, Waltham, MA, USA) and 5% CO_2_ at 37°C. To mimic the high-glucose environments in the pathogenesis of GDM, the cells were cultured in the high-glucose concentration medium with 25 mM glucose. The cells in normal controls group were cultured under the conditions with normal glucose (5 mM).

### 2.3. Cell Transfection

To achieve the *in vitro* manipulation of miR-136, human trophoblast cell lines HRT-8/SVneo and BeWo were transfected with miR-136 mimics, miR-136 inhibitors, or negative controls (mimic NC and inhibitor NC) (GenePharma, Shanghai, China) by Lipofectamine 2000 (Invitrogen, Carlsbad, CA, USA). In addition, an overexpression vector of E2F1 pcDNA3.1-E2F1 was synthesized by GenePharma (Shanghai, China) and was transfected into trophoblast cells using Lipofectamine 2000 (Invitrogen). The cell transfection experiment was performed according to the manufacturer's instruction. The subsequent cell analyses were carried out at 48 h posttransfection.

### 2.4. RNA Extraction and Quantitative Real-Time PCR (qRT-PCR)

By using TRIzol reagent (Invitrogen, Carlsbad, CA, USA), the total RNA in placental tissues and trophoblast cells was extracted. The total RNA in serum samples was extracted using TRIzol LS reagent (Invitrogen, Carlsbad, CA, USA) following the manufacturer's protocols. cDNA was conducted from RNA by reverse transcription using the PrimeScript RT reagent (TaKaRa, Shiga, Japan). The relative expression of targeted RNAs was evaluated using the qPCR, which was performed using the SYBR-Green I Master Mix kit (Invitrogen, Carlsbad, California, USA) and the 7300 Real-Time PCR System (Applied Biosystems, USA). In this experiment, amplification efficiency was more than 95%, and at least 3 replicates were set up. The primer sequences are listed in Table 1. The relative expression of miR-136 and mRNA of E2F1 was calculated using the 2^-*ΔΔ*Ct^ method and normalized to U6 and GAPDH, respectively.

### 2.5. CCK-8 Assay

After 48 h of cell transfection, trophoblast cells were seeded into 96-well plates, and cell proliferation assays were performed using a Cell Counting Kit-8 (CCK-8, Beyotime, Shanghai, China). The cell plates were cultured in an incubator at 37°C for 3 days, and CCK-8 was added at each well at different time points (24, 48, and 72 h). After 2 h of incubation, the optical density was measured at 450 nm on a microplate reader (Molecular Devises, CA, USA).

### 2.6. Luciferase Reporter Assay

This study found the putative binding site of miR-136 at the 3′-UTR of E2F1 according to the TargetScan (http://www.targetscan.org/vert_72/). A luciferase reporter assay was applied to confirm the interaction between miR-136 and E2F1. The wild-type (WT) and mutant-type (MUT) 3′-UTR sequences of E2F1 were combined into the luciferase reporter vector PMIR-RB-REPORT (RiboBio, Guangzhou, China). The recombinant vectors were cotranscribed into HRT-8/SVneo cells with miR-136 mimic, miR-136 inhibitor, or the NCs using Lipofectamine 2000 (Invitrogen) following the protocols of the manufacturer. The relative luciferase activity was detected using a Luciferase Reporter System (Promega, Wisconsin, USA) and normalized to Renilla luciferase activity.

### 2.7. Statistical Analysis

All the experiments and examinations were performed at least three times. Data in this study were expressed as the mean ± SD and analyzed using SPSS 22.0 (SPSS Inc., Chicago, IL) and GraphPad Prism 7.0 software (GraphPad Software, Inc., USA). Comparisons between groups were analyzed by Student's *t*-test or one-way ANOVA. The correlation between FBG levels and serum miR-136 was performed using the *χ*^2^ test. A *P* < 0.05 was considered to indicate a statistically significant difference.

## 3. Results

### 3.1. Baseline Characteristics of the Study Population

Table 2 lists the baseline characteristics of the 112 GDM patients and 58 healthy pregnant women included in this study, which showed that no statistical difference was observed between the two groups at age, body mass index (BMI), pregnancy week, and placental weight (all *P* > 0.05), and the GDM patients had significantly higher FBG compared with the health controls (*P* < 0.001).

### 3.2. Expression of miR-136 in Patients with GDM

**Figure 1:**
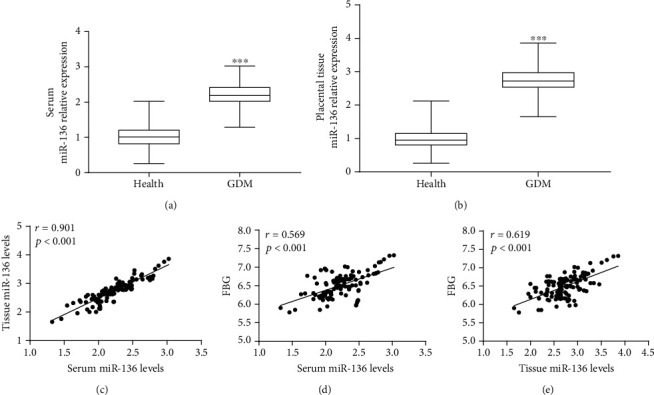
Expression of miR-136 in GDM patients and its diagnostic value evaluation. (a) GDM patients had higher serum miR-136 expression than the healthy controls (^∗∗∗^*P* < 0.001). (b) Expression of miR-136 in placental tissues was elevated in GDM patients compared with the healthy pregnancy women (^∗∗∗^*P* < 0.001). (c) Serum miR-136 was positively correlated with tissue miR-136 in GDM patients (*r* = 0.901, *P* < 0.001). (d) Serum miR-136 levels were positively correlated with patients' FBG (*r* = 0.569, *P* < 0.001). (e) A positive correlation was observed between tissue miR-136 and FBG in GDM patients (*r* = 0.619, *P* < 0.001).

Serum and placental tissue were collected from healthy controls and patients with GDM; qRT-PCR was used to detect and compare the expression of miR-136 in serum and placental tissue between two groups. The results are shown in Figure 1(a); the expression of miR-136 in serum of GDM was significantly higher than that of healthy control group (*P* < 0.001). Similarly, the expression of miR-136 in the placental tissue of the patient group was also higher than that of healthy pregnant women (*P* < 0.001, Figure 1(b)), and a positive correlation was found between the serum and placental tissue expression of miR-136 in patients with GDM (*r* = 0.901, *P* < 0.001; Figure 1(c)). Furthermore, the correlation of miR-136 with FBG was evaluated in GDM patients, and the results shown in Figures 1(d) and 1(e) revealed that both serum and placental tissue miR-136 levels were positively correlated with FBG in GDM women (both *P* < 0.001).

### 3.3. High-Glucose Treatment Inhibits Trophoblast Cell Proliferation but Enhances miR-136 Expression

**Figure 2:**
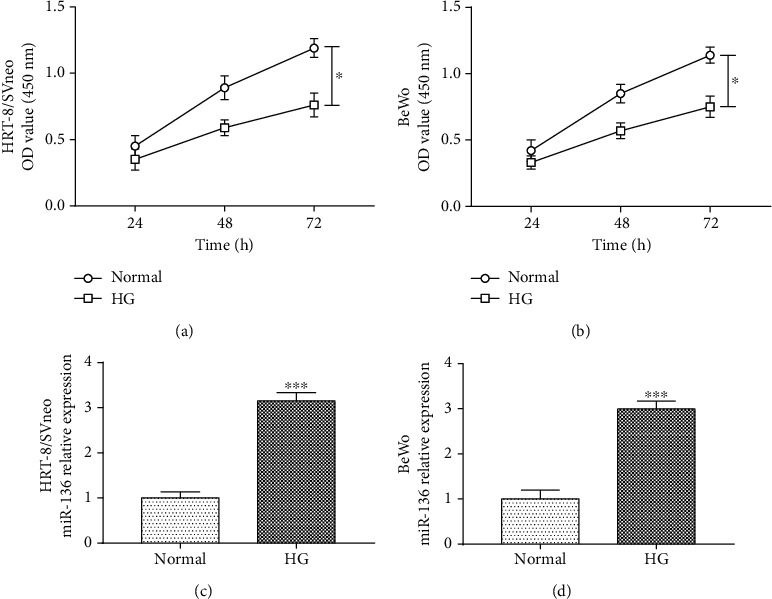
High-glucose treatment regulates trophoblast cell proliferation and miR-136 expression in HRT-8/SVneo and BeWo cells. (a, b) High glucose led to inhibited trophoblast cell proliferation (^∗^*P* < 0.05). (c, d) miR-136 expression was increased in both HRT-8/SVneo and BeWo cells under high-glucose conditions (^∗∗∗^*P* < 0.001). HG: high glucose.

High-glucose medium was applied for trophoblast cells incubation to mimic the high-glucose environment in the pathogenesis of GDM. As shown in Figures 2(a) and 2(b), the proliferation ability of the trophoblast cells in the high-glucose group was significantly inhibited compared with the cells in normal medium (all *P* < 0.05), which demonstrated that the GDM cell injury model was successfully constructed. In addition, the expression of miR-136 was expected to be promoted in both HRT-8/SVneo and BeWo cells under high-glucose conditions (both *P* < 0.001, Figures 2(c) and 2(d)).

### 3.4. Silencing of miR-136 Improves High-Glucose-Impaired Trophoblast Cell Proliferation

**Figure 3:**
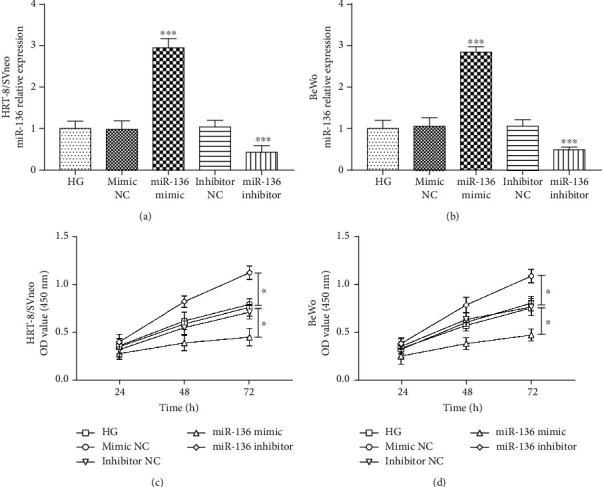
Reverse effect of miR-136 silencing on the impaired trophoblast cell viability resulted from high glucose in HRT-8/SVneo and BeWo cells. (a, b) miR-136 mimic increased, while miR-136 inhibitor inhibited the expression of miR-136 in trophoblast cells (^∗∗∗^*P* < 0.001). (c, d) The inhibiting effect of high glucose on trophoblast cell proliferation was enhanced by the overexpression of miR-136, but was reversed by the knockdown of miR-136 (^∗^*P* < 0.05). HG: high glucose.

We further analyzed the effect of miR-136 expression on cell proliferation activity by regulating the miR-136 level in trophoblast cells through a cell transfection method. The data in Figures 3(a) and 3(b) revealed that the expression of miR-136 was upregulated by miR-136 mimic, but was downregulated by the miR-136 inhibitor in both high-glucose-treated HRT-8/SVneo and BeWo cells (all *P* < 0.001). The CCK-8 assay results shown in Figures 3(c) and 3(d) demonstrated that the decreased trophoblast cell viability induced by high-glucose treatment was reversed by the overexpression of miR-136, while the knockdown of miR-136 could strengthen the high-glucose-induced cell proliferation impairment in both HRT-8/SVneo and BeWo cells (all *P* < 0.05).

### 3.5. Relationship between miR-136 and E2F1 in GDM

**Figure 4:**
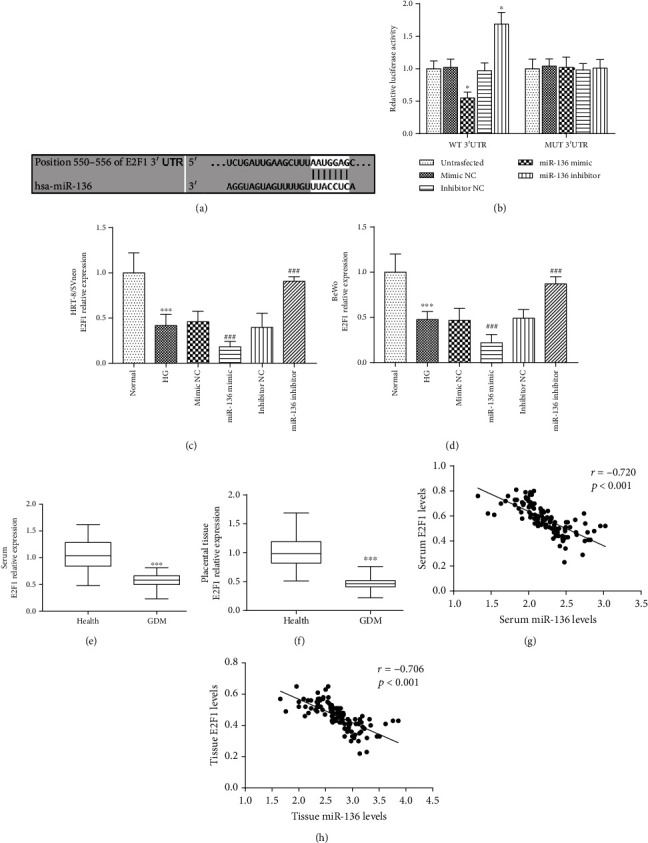
miR-136 directly regulates E2F1 in trophoblast cells and their negative correlation. (a) The putative binding site of miR-136 at the 3′-UTR of E2F1. (b) The relative luciferase activity was inhibited by the overexpression but enhanced by the knockdown of miR-136 in wild-type (WT) 3′UTR group (^∗^*P* < 0.05). (c, d) The expression of E2F1 was inhibited by high glucose, and this effect was strengthened by miR-136 overexpression, but was weakened by the silencing of miR-136 in both HRT-8/SVneo and BeWo cells (^∗∗∗^*P* < 0.001 vs. normal; ^###^*P* < 0.001 vs. HG). (e, f) The expression of E2F1 in serum and placental tissues was decreased in GDM patients compared with healthy controls (^∗∗∗^*P* < 0.001). (g, h) The levels of miR-136 were negatively correlated with E2F1 levels in GDM patients. HG: high glucose.

According to bioinformatics analysis, a putative binding site of miR-136 was found at the 3′-UTR of E2F1 (Figure 4(a)). The subsequent luciferase reporter assay results showed that the relative luciferase activity in the WT 3′-UTR group was significantly inhibited by the upregulation of miR-136, but was promoted by the reduction of miR-136 (all *P* < 0.05, Figure 4(b)). Furthermore, the relative mRNA expression of E2F1 in HRT-8/SVneo and BeWo cells was found to be inhibited by the high-glucose treatment (*P* < 0.001), and the overexpression of miR-136 in the cell model could inhibit E2F1, but the silencing of miR-136 led to the enhanced expression of E2F1 (all *P* < 0.001, Figures 4(c) and 4(d)). In enrolled pregnant women, we found that the mRNA expression levels of E2F1 in serum and placental tissues were downregulated in GDM women compared with healthy controls (both *P* < 0.001, Figures 4(e) and 4(f)) and were negatively correlated with miR-136 levels in GDM patients (both *P* < 0.001, Figures 4(g) and 4(h)).

### 3.6. E2F1 Reverses the Effect of miR-136 on Trophoblast Cell Proliferation

**Figure 5:**
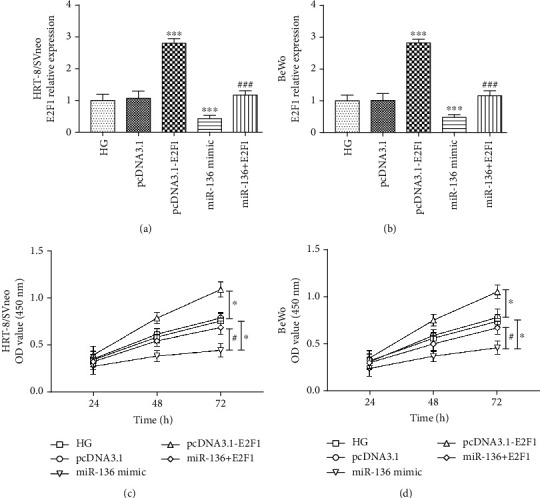
Effect of E2F1 on trophoblast cell proliferation. (a, b) The expression of E2F1 was upregulated by pcDNA.1-E2F1, and the inhibited E2F1 by miR-136 overexpression was abolished by pcDNA3.1-E2F1 in both HRT-8/SVneo and BeWo cells (^∗∗∗^*P* < 0.001 vs. HG; ^###^*P* < 0.001 vs. miR-136 mimic). (c, d) The inhibited trophoblast cell proliferation induced by the miR-136 overexpression was reversed by the upregulation of E2F1 in both HRT-8/SVneo and BeWo cells (^∗∗∗^*P* < 0.001 vs. HG; ^###^*P* < 0.001 vs. miR-136 mimic). HG: high glucose.

In the trophoblast injury cell model induced by high-glucose, the expression of E2F1 was overexpressed by pcDNA3.1-E2F1 (*P* < 0.001, Figures 5(a) and 5(b)). The cell proliferation of both HRT-8/SVneo and BeWo cells was promoted by the elevation of E2F1 (all *P* < 0.05, Figures 5(c) and 5(d)). In the cell model cotransfected with miR-136 mimic and pcDNA3.1-E2F1, the inhibited E2F1 by miR-136 overexpression was significantly elevated (*P* < 0.01, Figures 5(a) and 5(b)). Regarding the cell proliferation results, the overexpression of E2F1 could significantly reverse the inhibiting effect of miR-136 on cell proliferation in both HRT-8/SVneo and BeWo cells under high-glucose conditions (all *P* < 0.05, Figures 5(c) and 5(d)).

## 4. Discussion

Emerging evidence has reported the important role of miRNAs in the development and progression of GDM [19–21]. This study investigated the role of miR-136 in GDM, and the analysis results showed that the expression of miR-136 in both serum and placental tissues was significantly elevated and positively correlated with FBG levels in GDM patients. Furthermore, we found the increased expression of miR-136 in high-glucose-induced trophoblast cells, and the inhibition of miR-136 could alleviate the inhibited cell viability induced by high-glucose treatment. E2F1 was demonstrated to serve as a target gene of miR-136 in trophoblast cells, which was reduced in GDM patients and negatively correlated with miR-136. Additionally, we found that the upregulation of E2F1 could reverse the effects of miR-136 on trophoblast cell proliferation.

Some miRNAs have been found to be abnormally expressed in placental tissue, trophoblast cells, or human umbilical vein endothelial cells under high-glucose conditions [22–24]. Trophoblast cells are important cells involved in embryo implantation and functional placenta formation, which play pivotal roles in regulating the development of placenta [25]. During the progression of GDM, high-glucose environment could lead to the impairments in trophoblast cell function, contributing to rates of abortion and fetus malformation [26]. Therefore, exploring functional miRNAs that have regulatory potencies in trophoblast cell viability is of important value for the treatment of GDM. Zhou and his colleagues have documented the reduced miR-132 in GDM patients could regulate trophoblast cell proliferation, which indicated the therapeutic potential of miR-132 for GDM [27]. Peng et al. also investigated the deregulated miR-137 in GDM and demonstrated that miR-137 was involved in the regulation of trophoblast cell viability through the PRKAA1/IL-6 pathway [28]. In this study, we focused on the role of miR-136 in GDM, which has been reported to aberrantly express in GDM by Gillet et al. [12]. We found the significant upregulation in miR-136 expression in both serum and placental tissues in GDM patients compared with healthy controls, and miR-136 expression was positively correlated with FBG levels in GDM patients, which indicated that miR-136 might be involved in the development of GDM.

Accumulating studies have highlighted the diagnostic potential of aberrant miRNAs in various human diseases [29, 30]. The clinical significance of miR-136 in disease diagnosis has also been reported in some diseases. For example, Motawi et al. have demonstrated that miR-136 could be used to distinguish patients with eclampsia as a candidate diagnostic biomarker [31]. The expression of miR-136 in patients infected with hepatitis C was elevated, which could be used to diagnose hepatitis C-infected liver disease patients [32]. Given the abnormal expression of miR-136 in GDM, we suspected that the significantly increased expression of miR-136 in serum samples might also serve as a candidate biomarker for GDM early diagnosis. Further studies having a larger population are needed to confirm the clinical significance of miR-136 in GDM.

GDM is characterized by high-blood glucose, which leads to the impairments in trophoblast cell function. Trophoblast cell viability can be suppressed by high glucose, but the understanding about the underlying mechanisms remains limited. A previous study investigated the effects of high glucose on trophoblast BeWo cells and stated that the inhibited cell proliferation induced by high glucose might be related with dysregulation of heparin-binding-EGF (HB-EGF), which is a known survival factor for trophoblasts [33, 34]. In another study regarding the differentially expressed miRNA in GDM, high glucose induced the upregulated miR-137 in trophoblasts, and the miR-137/PRKAA1/IL-6 axis was proposed to mediate the inhibitory effects of high glucose on trophoblast cell proliferation [28]. This study used high-glucose medium to induce an injury trophoblast cell model, and the cell proliferation was expected to be inhibited. Of note, the expression of miR-136 was significantly upregulated in trophoblast cells treated with high-glucose stimulation. Previous studies have reported the regulatory effect of miR-136 on cell proliferation in different kinds of cell, such as keratinocytes [35] and osteosarcoma cells [36]. Nevertheless, whether miR-136 could influence trophoblast cell proliferation remains unclear. In this study, we observed that the silencing of miR-136 could reverse the inhibiting effect of high glucose on trophoblast cell proliferation, while the overexpression of miR-136 strengthened the effect of high glucose on trophoblast cell proliferation, which implied us that high glucose might impair trophoblast cell viability by increasing miR-136. Thus, the methods to reduce miR-136 might be novel therapeutic approaches for GDM by improving trophoblast cell proliferation. However, the specific potential mechanisms involved in the regulation of miR-136 by glucose remain unclear. Some molecules have been reported to regulate miRNA and also served as targets of miRNAs, leading to a feedback loop and regulation in disease progression [37]. In addition, some competing endogenous RNAs (ceRNAs) are also related with the differential levels of miRNA during pathological changes. LINC00657, circular RNA hsa_circ_0023404, and LINC01116 have been reported to sponge miR-136, then regulate cell proliferation [38–40]. The mechanisms underlying the upregulation of miR-136 under high-glucose conditions need to be investigated in future studies.

This study predicted the potential targets of miR-136 and demonstrated that E2F1 was a potential target gene of miR-136. E2F1 is the first identified member of the E2F family, which serves as an important regulator of cell cycle [14]. The interaction between miR-136 and E2F1 has been documented in cervical carcinoma, and the miR-136/E2F1 axis played a functional role in the regulation of tumor cell proliferation [13]. This study demonstrated the inhibitory effect of miR-136 on E2F1 in trophoblast cells under high-glucose conditions, and the expression of E2F1 in GDM patients was found to be negatively correlated with miR-136, which indicated the close relationship between miR-136 and E2F1 in the progression of GDM. Furthermore, in trophoblast cells with high-glucose stimulation, the inhibiting effect of miR-136 overexpression was significantly reversed by the upregulation of E2F1, suggesting that the regulatory effect of miR-136 on trophoblast cell viability might be mediate by E2F1. Collectively, high glucose induced increased miR-136 in trophoblast cells, leading to inhibited cell proliferation by suppressing E2F1.

In summary, this study demonstrated that the expression of miR-136 was significantly increased in both serum and placental tissues in GDM patients. High-glucose treatment induces inhibited trophoblast cell proliferation, and this effect may be achieved by increasing miR-136 and decreasing E2F1. The miR-136/E2F1 axis provides a novel insight into the pathogenesis involving the injury induced by high glucose in GDM, and novel GDM therapeutic approaches may be developed by regulating this axis. This study investigates the functional role of miR-136 only by *in vitro* assay, which is a limitation of this study, and further investigations should be carried out using *in vivo* analysis.

## Figures and Tables

**Table 1:** Primer sequences for qRT-PCR.

Name	Primer sequence (5′-3′)
miR-136	Forward: GCCGAGACTCCATTTGTTT
	Reserved: CTCAACTGGTGTCGTGGA

E2F1	Forward: CCGTGGACTCTTCGGAGAAC
	Reserved: ATCCCACCTACGGTCTCCTC

U6	Forward: CTCGCTTCGGCAGCACA
	Reserved: AACGCTTCACGAATTTGCGT

GAPDH	Forward: GACTCATGACCACAGTCCATGC
	Reserved: AGAGGCAGGGATGATGTTCTG

**Table 2:** Baseline characteristics of the study population.

Characteristics	Health	GDM	*P* value
Age (years)	31.14 ± 3.94	31.59 ± 3.93	0.480
BMI (kg/m^2^)	22.22 ± 3.59	23.35 ± 3.56	0.055
Pregnancy weeks (weeks)	24.47 ± 2.15	24.85 ± 1.69	0.206
Placental weight (kg)	0.56 ± 0.11	0.58 ± 0.16	0.298
FBG (mM/L)	4.39 ± 0.33	6.51 ± 0.34	<0.001

GDM: gestational diabetes mellitus; BMI: body mass index; FBG: fasting blood glucose. Data were expressed as the mean ± SD.

## Data Availability

All data analyzed during this study are included in this published article.

## Abbreviations

GDM: Gestational diabetes mellitus
miRNAs: MicroRNAs
NC: Negative control
qRT-PCR: Quantitative real-time PCR
CCK-8: Cell Counting Kit-8
BMI: Body mass index
FBG: Fasting blood glucose
ROC: Receiver operating characteristic curve
AUC: Area under the curve
E2F1: E2F transcription factor 1.
